# Elevated microsatellite instability at selected tetranucleotide (EMAST) repeats in gastric cancer: a distinct microsatellite instability type with potential clinical impact?

**DOI:** 10.1002/cjp2.257

**Published:** 2022-01-31

**Authors:** Anna‐Lina Herz, Sarah Wisser, Meike Kohlruss, Julia Slotta‐Huspenina, Moritz Jesinghaus, Bianca Grosser, Katja Steiger, Alexander Novotny, Alexander Hapfelmeier, Thomas Schmidt, Matthias M Gaida, Wilko Weichert, Gisela Keller

**Affiliations:** ^1^ Institute of Pathology, TUM School of Medicine Technical University of Munich Munich Germany; ^2^ Institute of Pathology University Hospital Marburg Marburg Germany; ^3^ Institute of Pathology and Molecular Diagnostics University Hospital Augsburg Augsburg Germany; ^4^ German Cancer Consortium [DKTK], Partner Site Munich Institute of Pathology Munich Germany; ^5^ Department of Surgery, TUM School of Medicine Technical University of Munich Munich Germany; ^6^ Institute for AI and Informatics in Medicine Technical University of Munich Munich Germany; ^7^ Institute of General Practice and Health Services Research, TUM School of Medicine Technical University of Munich Munich Germany; ^8^ Department of Surgery University of Heidelberg Heidelberg Germany; ^9^ Department of Surgery Universitätsklinikum Köln Köln Germany; ^10^ Institute of Pathology University of Heidelberg Heidelberg Germany; ^11^ Institute of Pathology University Medical Center Mainz Mainz Germany

**Keywords:** EMAST, microsatellite instability, gastric adenocarcinoma, neoadjuvant chemotherapy, prognosis

## Abstract

We investigated the clinical impact of elevated microsatellite instability at selected tetranucleotide (EMAST) repeats in the context of neoadjuvant chemotherapy (CTx) in gastric/gastro‐oesophageal adenocarcinomas. We analysed 583 resected tumours (272 without and 311 after CTx) and 142 tumour biopsies before CTx. If at least two or three of the five tetranucleotide repeat markers tested showed instability, the tumours were defined as EMAST (2+) or EMAST (3+), respectively. Expression of mismatch repair proteins including MSH3 was analysed using immunohistochemistry. Microsatellite instability (MSI) and Epstein–Barr virus (EBV) positivity were determined using standard assays. EMAST (2+) and (3+) were detected in 17.8 and 11.5% of the tumours, respectively. The frequency of EMAST (2+) or (3+) in MSI‐high (MSI‐H) tumours was 96.2 or 92.5%, respectively, demonstrating a high overlap with this molecular subtype, and the association of EMAST and MSI status was significant (each overall *p* < 0.001). EMAST (2+ or 3+) alone in MSI‐H and EBV‐negative tumours demonstrated only a statistically significant association of EMAST (2+) positivity and negative lymph node status (42.3% in EMAST (2+) and 28.8% in EMAST negative, *p* = 0.045). EMAST alone by neither definition was significantly associated with overall survival (OS) of the patients. The median OS for EMAST (2+) patients was 40.0 months (95% confidence interval [CI] 16.4–63.6) compared with 38.7 months (95% CI 26.3–51.1) for the EMAST‐negative group (*p* = 0.880). The median OS for EMAST (3+) patients was 46.7 months (95% CI 18.2–75.2) and 38.7 months (95% CI 26.2–51.2) for the negative group (*p* = 0.879). No statistically significant association with response to neoadjuvant CTx was observed (*p* = 0.992 and *p* = 0.433 for EMAST (2+) and (3+), respectively). In conclusion, our results demonstrate a nearly complete intersection between MSI‐H and EMAST and they indicate that EMAST alone is not a distinct instability type associated with noticeable clinico‐pathological characteristics of gastric carcinoma patients.

## Introduction

Elevated microsatellite instability at selected tetranucleotide (EMAST) repeats is a type of microsatellite instability (MSI) occurring preferentially at microsatellite markers with tetranucleotides as the repeat unit, and dysfunction specifically of the mismatch repair (MMR) protein MSH3 has been discussed as a cause for EMAST [1, 2]. The classical term MSI usually refers to instability determined at mono‐ and dinucleotide repeat markers using standardised panels comprising in general five microsatellite markers [3, 4]. If at least two of the five tested markers show instability, the tumour is classified as MSI‐high (MSI‐H) and if only one marker is unstable as MSI‐low (L). MSI‐H is due to defects in the DNA MMR proteins, MLH1, MSH2, MSH6, or PMS2, which normally correct the frequent replication errors occurring at short repetitive microsatellite sequences. MSI‐H has been identified as an important, distinct molecular subtype in 10–22% of gastric carcinomas (GC) and an association with specific clinical characteristics such as intestinal histotype, distal tumour location, female sex, and older age has been demonstrated [5, 6, 7, 8, 9]. Of note, MSI‐H has been related to increased survival in the majority of studies [7, 10]. For patients treated with neoadjuvant chemotherapy (CTx), controversial results have been reported, as a negative prognostic effect of MSI‐H as well as an association of MSI‐H with favourable prognosis, specifically for females, have been shown in this therapeutic setting [8, 9, 11].

EMAST has been described in several tumour entities including GC, with broad variation in incidence ranging from 8 to 60% in gastrointestinal tumours, which may be related in part to the lack of a standardised definition and marker panels used for the determination of EMAST [12, 13, 14, 15, 16, 17, 18, 19]. A partial overlap of EMAST with MSI‐H and also with the MSI‐L phenotype has been reported, and it is not clear if EMAST differentiates MSI‐H tumours into two subgroups with potential consequences for treatment with immune therapy and if EMAST alone represents a unique molecular subclass [15, 17, 18, 19, 20]. Furthermore, knowledge about EMAST particularly in GC is limited.

In recent studies, we performed a comprehensive molecular characterisation in large GC cohorts, including determination of the molecular subgroups MSI and Epstein–Barr virus positive (EBV+), encompassing patients treated with and without neoadjuvant platinum/5‐fluorouracil (5‐FU)‐based CTx [8, 9, 21]. MSI and EBV‐positive tumours are two of the molecular subtypes described by the Cancer Genome Atlas Consortium (TCGA) [5]. They are characterised by distinct genetic and clinico‐pathological features and can be determined by standardised assays [5, 7, 8].

In the present study, we aimed to clarify if EMAST delineates tumours with specific properties in the context of these well‐defined molecular subgroups, analysing our cohorts for EMAST in relation to MSI and EBV status. In a second step, we asked if EMAST alone represents a unique instability type associated with specific clinical, prognostic, and predictive characteristics of patients with EBV‐ and MSI‐H‐negative tumours. Furthermore, we investigated whether EMAST may be associated with aberrant expression of MMR proteins with particular emphasis on the expression of MSH3.

## Material and methods

### Patients

Surgically resected gastric adenocarcinomas including tumours of the gastro‐oesophageal junction (AEG II and AEG III according to Siewert and Stein [22]) from 583 patients, who were treated with (*n* = 311) or without (*n* = 272) neoadjuvant CTx between 2001 and 2013 at the Department of Surgery of the University of Heidelberg and between 2001 and 2012 at the Technical University of Munich, were included in the study. Characteristics of the 583 patients are summarised in Table 1.

**Table 1:** Patient's characteristics

		Resected tumours without and after neoadjuvant CTx		Biopsies before neoadjuvant CTx	
Category	Value	*n*	%	*n*	%
**Cases**	Total	583	100	142	100
**Age (years)**	Median	64.6		61.4	
	Range	29.2–90.9		23.3–79.1	
**Follow‐up period (months)**	Median	57.8		69.6	
	95% CI	53.3–62.4		61.1–78.2	
**Overall survival (months)**	Median	44.4		48.1	
	95% CI	29.5–59.3		26.3–69.9	
**Number of events**		276	47.3	77	54.2
**Sex**	Male	433	74.3	108	76.1
	Female	150	25.7	34	23.9
**Tumour localisation**	Proximal	285	48.9	100	70.4
	Middle	144	24.7	23	16.2
	Distal	123	21.1	13	9.2
	Total/linitis	27	4.6	6	4.2
	N/A	4	<1	–	–
**Laurén classification**	Intestinal	326	55.9	72	50.7
	Non‐intestinal	257	44.1	70	49.3
**Tumour grade**	G1/2	118	20.2	33	23.2
	G3/4	384	659	109	76.8
	N/A	81	13.9	–	–
**Clinical tumour stage**	cT2	132	22.6	7	4.9
	cT3/cT4	449	77.0	129	90.8
	N/A	2	<1	6	4.2
**(y)pT** *	(y)pT0	–	–	9	6.3
	(y)pT1	53	9.1	12	8.5
	(y)pT2	73	12.5	19	13.4
	(y)pT3	308	52.8	81	57.0
	(y)pT4	149	25.6	19	13.4
	N/A	–	–	2	1.4
**(y)pN** *	Negative	180	30.9	61	43.0
	Positive	403	69.1	79	55.6
	N/A	–	–	2	1.4
**Metastasis status**	No	503	86.3	96	67.6
	Yes	80	13.7	44	31.0
	N/A	–	–	2	1.4
**Resection category**	R0	443	76.0	116	81.7
	R1	140	24.0	24	16.9
	N/A	–	–	2	1.4
**Neoadjuvant CTx**	No	272	46.7	–	–
	Yes	311	53.3	142	100
**Tumour regression grade** ^†^	TRG1	–	–	45	31.7
	TRG2	147	25.2	34	23.9
	TRG3	164	28.1	63	44.4
	Total^†^	311	53.3	142	100
**Response**	TRG1	–	–	45	31.7
	TRG2/3	311	100	97	68.3
**MSI status**	MSS	502	86.1	120	84.5
	MSI‐L	28	4.8	7	4.9
	MSI‐H	53	9.1	15	10.6
**EBV status**	EBV negative	563	96.6	137	96.5
	EBV positive	20	3.4	5	3.5
**EMAST status**	Negative	479	82.2	111	78.2
	Positive 2+	104	17.8	31	21.8
**EMAST status**	Negative	516	88.5	120	84.5
	Positive 3+	67	11.5	22	15.5

* TNM classification of malignant tumours according to the Seventh Edition of the UICC.

^**†**^ TRG corresponded only to patients with tumours treated with neoadjuvant CTx.

Pretherapeutic biopsies from 142 patients with advanced carcinomas who were treated with platinum/5‐FU‐based neoadjuvant CTx were also analysed. The biopsies were considered as a separate cohort, as it allows the evaluation of EMAST in association with response to CTx including patients with complete tumour regression. Characteristics of the 142 patients were essentially as described previously and for the sake of completeness included in Table 1 [8].

Inclusion criterion for all patients in the present study was the successful determination of EMAST from tumours with known MSI and EBV status described in former studies [8, 21].

**Figure 1:**
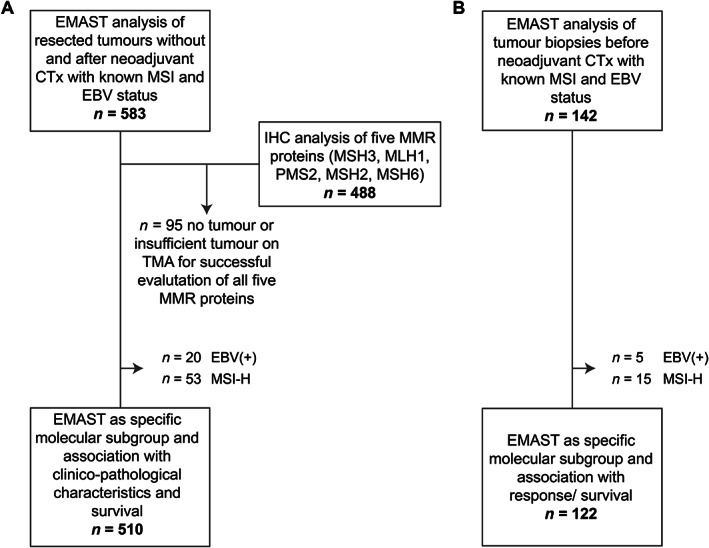
Flow chart diagram of patient and specimen inclusion. The total number of patients included in EMAST analysis is shown for (A) the resected tumour cohort treated with or without CTx and (B) the tumour biopsy cohort before neoadjuvant CTx. IHC, immunohistochemistry; TMA, tissue microarray.

An overview of patient enrolment in the present study is shown in Figure 1. Our study has to be considered as a retrospective exploratory analysis.

### CTx and surgery

The indication for neoadjuvant CTx was as described in detail previously [23, 24]. In brief, eligibility included the presence of locally advanced adenocarcinoma (cT3‐T4, any N, cM0 by endoscopy, endoluminal ultrasound, and CT scan). Patients who received neoadjuvant CTx were treated with platinum/5‐FU‐based regimens as detailed in supplementary material, Table S1. All surgical procedures included an abdominal D2 lymphadenectomy and were described in detail elsewhere [8].

### Response evaluation

Response to neoadjuvant CTx was determined histopathologically and was classified into three tumour regression grades (TRG) according to the Becker classification [23], TRG1, TRG2, and TRG3, which corresponded to <10, 10–50, and >50% of residual tumour cells/tumour bed, respectively. This classification system has demonstrated prognostic relevance and accordingly patients with TRG1 were classified as responders and patients with TRG2 and TRG3 as non‐responders [23].

### Follow‐up and OS

Follow‐up was performed as described previously [8]. Overall survival (OS) was defined as the time between the date of surgery and death by any cause.

### Ethics statement

The study was approved by the local Institutional Review Board at the Technical University Munich (342/19 S‐SR) and at the University of Heidelberg (reference: 301/2001). All experiments were performed in accordance with the Declaration of Helsinki and informed consent was obtained according to institutional regulations.

### EMAST analysis and definition

DNA from formalin‐fixed paraffin‐embedded (FFPE) normal and tumour tissues was isolated after manual microdissection as described previously and was used for analysis [8, 9]. Samples with a tumour cell content of at least 10% were included for MSI analysis according to the described limit of detection for MSI [25].

EMAST was analysed using the five tetranucleotide repeats, D20S85, D20S82, D9S242, D8S321, and MYCL1, which are frequently used for the determination of this type of MSI [14]. Primer sequences are shown in supplementary material, Table S2. A multiplex PCR with fluorescence‐tagged primers was performed using the Type‐it Microsatellite PCR kit (Qiagen, Hilden, Germany) on non‐tumour or tumour DNA and is described in detail in Supplementary materials and methods.

EMAST positivity was defined as at least two of the five markers or ≥40% of the markers showed an additional allele in the tumour compared to DNA from non‐tumorous tissue and was designated EMAST (2+). In addition, we tested a more stringent classification and defined EMAST positivity if at least three of the five markers or ≥50% of the markers showed instability and designated it as EMAST (3+).

### MSI analysis

Tumours had been analysed for MSI previously using the Bethesda panel encompassing two mononucleotide repeats (BAT25 and BAT26) and three dinucleotide repeats (D2S123, D5S346, and D17S250) [3, 8]. Tumours with only instability at two dinucleotides were additionally analysed using three mononucleotides (NR‐21, NR‐24, and NR‐27). Tumours were classified as MSI‐H if at least two markers of the Bethesda panel were unstable including at least one unstable mononucleotide marker or if at least two of the five mononucleotide markers showed instability essentially as described [9].

Tumours showing instability only at one marker or only at two dinucleotide markers of the Bethesda panel were classified as MSI‐L. Microsatellite stable (MSS) tumours did not show any instability.

### Tissue microarray and immunohistochemical analysis

FFPE tumour samples were assembled into tissue microarrays using a Tissue Microarrayer (Beecher Instruments, Sun Prairie, Wisconsin, USA) with a core size of 0.6 mm. At least three cores from the invasion front and the tumour core region were selected from tumour areas marked by a pathologist as described previously [21]. Immunohistochemistry was performed on a BenchMark XT automated stainer (Ventana, Roche, Mannheim, Germany). Expression of the MMR proteins, MLH1, PMS2, MSH2, MSH6, and MSH3, was analysed using the antibodies and dilutions as listed in supplementary material, Table S3.

Scoring for the expression of MMR proteins was performed using stromal or normal epithelial cells with nuclear staining as internal positive controls. Scoring for expression of MSH2, MSH6, MLH1, and PMS2 was as follows: absent or nuclear staining in less than 10% of the tumour cells was defined as loss or strongly reduced expression. Expression in more than 10% of the tumour cells was defined as normal. Expression of MSH3 was evaluated using a three‐tiered scoring system and defined as loss or strongly reduced if <10%, moderate if 10–50%, and strong if >50% of the tumour cells showed nuclear staining. Scoring of MMR protein expression was performed blinded to clinical outcome. A subset of the tumours were scored by a second observer for inter‐observer reproducibility assessment. All tumours with aberrant or discordant scoring by two observers were re‐evaluated and reviewed together with an expert gastrointestinal pathologist (JS‐H or MJ) until a final consensus was reached.

### Statistical analysis

The distribution of continuous data is presented by median and range. Categorical data are described by absolute and relative frequencies. Chi‐squared tests or Fisher's exact tests were used for hypothesis testing of differences between the relative frequencies. Kaplan–Meier estimates of survival probabilities were compared by log‐rank tests. Statistical analyses were performed using SPSS, Version 25 (IBM Corp., Armonk, NY, USA). Exploratory 5% significance levels (two‐tailed) were used for hypothesis testing.

## Results

### Frequency of EMAST, MSI, and EBV in the resected tumours

**Figure 2:**
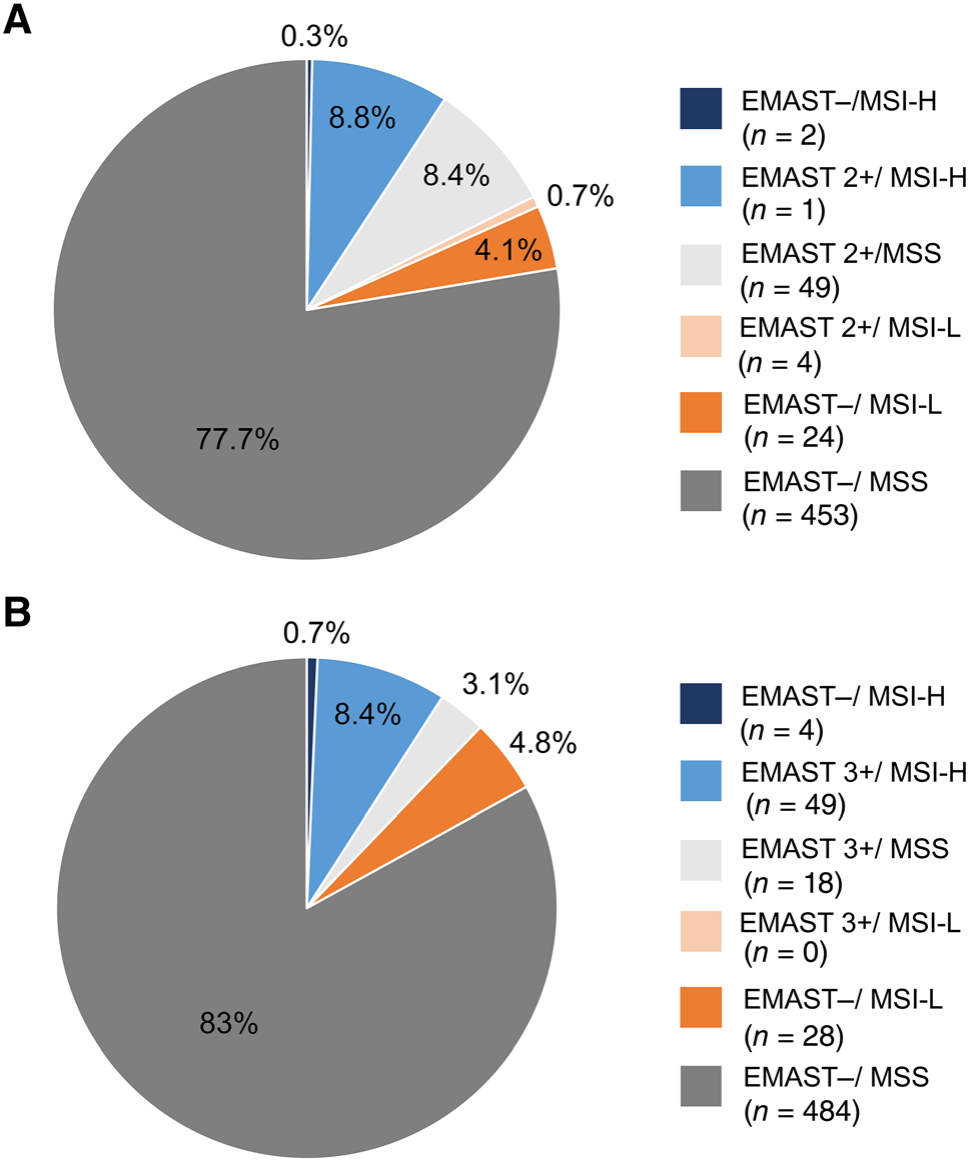
Distribution of instability types. EMAST and MSI status for the resected tumour cohort according to (A) EMAST definition 2+ and (B) 3+.

EMAST (2+) was detected in 104 of the 583 (17.8%) resected tumours. Considering the more stringent classification, EMAST (3+) was found in 67 (11.5%) of them. Regarding MSI in this study population, 53 (9.1%) tumours were MSI‐H, 28 (4.8%) were MSI‐L, and 502 (86.1%) were MSS. Results are summarised in Table 1. Taking into account the overlap between the instability types among the 583 tumours, we observed 51 (8.8%) EMAST (2+)/MSI‐H, 4 (0.7%) EMAST (2+)/MSI‐L, and 49 (8.4%) EMAST (2+)/MSS tumours. With respect to the EMAST (3+) definition, there were 49 (8.4%) EMAST (3+)/MSI‐H and 18 (3.1%) EMAST (3+)/MSS tumours. An overview of the distribution of the various instability frequencies is shown in Figure 2. In relation to EBV, 20 of 583 (3.4%) tumours were EBV positive. Results are included in Table 1.

### EMAST and correlation with MSI and EBV‐positive subgroups in the resected tumours

The association of EMAST (2+) and of EMAST (3+) with MSI status was statistically significant (each overall *p* < 0.001). In particular, 51 of 53 (96.2%) MSI‐H tumours, 4 of 28 (14.3%) MSI‐L tumours, and 49 of 502 (9.8%) MSS tumours were positive for EMAST (2+). Regarding EMAST (3+), 49 of the 53 (92.5%) MSI‐H tumours, none of the 28 MSI‐L tumours, and 18 of 502 (3.6%) MSS tumours were positive for EMAST. Results are summarised in Table 2. Thus, the observed significant associations were mainly due to the high overlap between MSI‐H and EMAST positivity and rather low frequencies of EMAST‐positive tumours in either definition in the MSI‐L and the MSS groups, indicating an overall strong positive association of EMAST with the MSI‐H, but not with the MSI‐L phenotype.

**Table 2:** Association of EMAST status and MSI and EBV status in the resected tumour cohort.

		MSI‐H (*n* = 53)	MSI‐L (*n* = 28)	MSS (*n* = 502)	*P* value*	EBV (−) (*n* = 563)	EBV (+) (*n* = 20)	*P* value*
**EMAST**	2+	51 (96.2)	4 (14.3)	49 (9.8)	<0.001	103 (18.3)	1 (5)	0.228
	−	2 (3.8)	24 (85.7)	453 (90.2)		460 (81.7)	19 (95)	
**EMAST**	3+	49 (92.5)	0 (0)	18 (3.6)	<0.001	67 (11.9)	0 (0)	0.151
	−	4 (7.5)	28 (100)	484 (96.4)		496 (88.1)	20 (100)	

* Fisher's exact or chi‐squared test.

Regarding EBV, no statistically significant association of EBV positivity with EMAST (2+) or EMAST (3+) was found (*p* = 0.228 and *p* = 0.151, respectively). Results are included in Table 2.

### EMAST and MSI and correlation with expression of the MMR proteins MSH3, MLH1, PMS2, MSH2, and MSH6

We analysed EMAST and MSI for aberrant expression of MMR proteins including MSH3, MLH1, MSH2, MSH6, and PMS2 in the resected tumours. Complete immunohistochemical results of all five MMR proteins and the molecular data were available for a subset of 488 tumours.

Regarding MSH3, only 7 of 488 (1.4%) tumours showed reduced expression, 79 (16.2%) showed moderate expression, and 402 (82.4%) showed strong expression (Table 3).

**Table 3:** Association of EMAST/MSI status and MSH3 expression.

		MSH3 expression						
Status		Reduced (<10%), *n* = 7	Moderate (10–50%), *n* = 79	Strong (>50%), *n* = 402	*P* value*	Reduced/moderate (≤50%), *n* = 86	Strong (>50%), *n* = 402	*P* value*
**EMAST**	−	6 (85.7)	57 (72.2)	332 (82.6)	0.092	63 (73.3)	332 (82.6)	0.046
	2+	1 (14.3)	22 (27.8)	70 (17.4)		23 (26.7)	70 (17.4)	
**EMAST**	−	6 (85.7)	63 (79.7)	358 (89.1)	0.072	69 (80.2)	358 (89.1)	0.025
	3+	1 (14.3)	16 (20.3)	44 (10.9)		17 (19.8)	44 (10.9)	
**MSI**	MSS	5 (71.4)	65 (82.3)	346 (86.1)	0.358	70 (81.4)	346 (86.1)	0.506
	MSI‐L	1 (14.3)	4 (5.1)	20 (5.0)		5 (5.8)	20 (5.0)	
	MSI‐H	1 (14.3)	10 (12.6)	36 (8.9)		11 (12.8)	36 (8.9)	

* Fisher's exact or chi‐squared test.

The distribution of these three MSH3 expression groups was not statistically significantly different among the EMAST (2+) or EMAST (3+) and EMAST‐negative tumours (*p* = 0.092 and *p* = 0.072, respectively). However, combining the tumours with reduced and moderate expression into one group, a significant association of lower MSH3 expression with EMAST (2+) and (3+) was found. Considering the EMAST (2+) category, 23 of 86 (26.7%) tumours with reduced/moderate expression compared to 70 of 402 (17.4%) tumours with strong MSH3 expression were EMAST (2+) positive (*p* = 0.046). Regarding EMAST (3+), 17 of 86 (19.8%) with reduced/moderate expression and 44 of 358 (10.9%) with strong MSH3 expression were EMAST (3+) positive (*p* = 0.025) (Table 3).

No significant association of MSH3 expression was found with MSI‐H, MSI‐L, or MSS tumours comparing either the three‐tiered (*p* = 0.358) or the two‐tiered (*p* = 0.506) MSH3 classification system. All results are summarised in Table 3.

With respect to the expression of the four ‘classical’ MMR proteins, all 46 MSI‐H tumours in this study population showed loss of expression of two or one of the MMR proteins (combined loss of MLH1/PMS2: *n* = 43; combined loss of MSH2/MSH6 *n* = 1; isolated loss of MSH6: *n* = 1; isolated loss of PMS2: *n* = 1). In addition, isolated loss of MSH6 was found in one MSS/MSI‐L and EMAST‐negative tumour. No aberrant expression of these MMR proteins was found in sole EMAST‐positive tumours. Results are summarised in supplementary material, Table S4.

### EMAST and association with clinico‐pathological characteristics and patient survival

To analyse if isolated EMAST positivity represents a unique molecular subgroup associated with specific clinico‐pathological characteristics or patient survival, we excluded MSI‐H and EBV+ tumours and compared the isolated EMAST (2+ and 3+)‐positive with the respective EMAST‐negative group.

No statistically significant association with specific patient characteristics such as age or sex, histological tumour type, tumour localisation, or TNM status was found, apart from an association of EMAST (2+) tumours with negative lymph node status (22 of 52 [42.3%] in EMAST (2+) and 132 of 458 [28.8%] in EMAST negative, *p* = 0.045).

Furthermore, a higher frequency of EMAST‐positive tumours was found in the patient group not treated with CTx. In the EMAST (2+)‐positive group, 31 of 52 (65.6%) patients were not treated with CTx compared to 204 of 458 (44.5%) in the EMAST‐negative group (*p* = 0.039). In the EMAST (3+)‐positive group, 13 of 18 (75%) patients were not treated with CTx compared to 222 of 492 (45.1%) in the negative group (*p* = 0.023). Results are summarised in Table 4.

**Table 4:** EMAST as specific molecular group and association with clinical‐pathological characteristics

		Resected tumours without or after neoadjuvant CTx*					
		EMAST−	EMAST 2+		EMAST−	EMAST 3+	
Category	Value	*n* (%)	*n* (%)	*P* value^†^	*n* (%)	*n* (%)	*P* value^†^
**Cases**	Total	458 (100)	52 (100)		492 (100)	18 (100)	
**Age (years)**	Median	64.4	64.0		64.4	64.1	
	Range	30.2–90.9	35.4–88.3		30.2–90.9	35.4–76.7	
**Follow‐up period (months)**	Median	57.9	48.8		57.9	44.4	
	95% CI	52.5–63.3	24.7–72.9		53.1–62.8	20.1–68.7	
**Overall survival (months)**	Median	38.7	40.0		38.7	46.7	
	95% CI	26.3–51.1	16.4–63.6		26.2–51.2	18.2–75.2	
**Number of events**		228 (49.8)	23 (44.2)		243 (49.4)	8 (44.4)	
**Gender**	Male	340 (74.2)	40 (76.9)	0.673	369 (75)	11 (61.1)	0.184
	Female	118 (25.8)	12 (23.1)		123 (25)	7 (38.9)	
**Tumour localisation**	Proximal	226 (49.4)	34 (65.4)	0.153	248 (50.4)	12 (66.6)	0.356
	Middle	110 (24.0)	9 (17.3)		115 (23.4)	4 (22.2)	
	Distal	95 (20.7)	6 (11.5)		100 (20.3)	1 (5.6)	
	Total/linitis	23 (5.0)	3 (5.8)		25 (5.1)	1 (5.6)	
	N/A	4 (<1)	–		4 (<1)	–	
**Laurén classification**	Intestinal	248 (54.1)	32 (61.5)	0.310	269 (54.7)	11 (61.1)	0.590
	Non‐intestinal	210 (45.9)	20 (38.5)		223 (45.3)	7 (38.9)	
**Tumour grade**	G1/2	92 (20.1)	15 (28.8)	0.267	100 (20.3)	7 (38.8)	0.096
	G3/4	301 (65.7)	34 (65.4)		325 (66.1)	10 (55.6)	
	N/A	65 (14.2)	3 (5.8)		67 (13.6)	1 (5.6)	
**Clinical tumour stage**	cT2	105 (22.9)	14 (26.9)	0.474	116 (23.6)	3 (16.7)	0.567
	cT3/cT4	352 (76.9)	37 (71.2)		375 (76.2)	14 (77.7)	
	N/A	1 (<1)	1 (1.9)		1 (<1)	1 (5.6)	
**(y)pT** ^‡^	(y)pT1	41 (8.9)	7 (13.5)	0.725	48 (9.8)	–	0.570
	(y)pT2	56 (12.3)	7 (13.5)		61 (12.4)	2 (11.1)	
	(y)pT3	240 (52.4)	26 (50.0)		254 (51.6)	12 (66.7)	
	(y)pT4	121 (26.4)	12 (23.0)		129 (26.2)	4 (22.2)	
**(y)pN** ^‡^	Negative	132 (28.8)	22 (42.3)	0.045	149 (30.3)	5 (27.8)	0.820
	Positive	326 (71.2)	30 (57.7)		343 (69.7)	13 (72.2)	
**Metastasis status**	No	386 (84.3)	47 (90.4)	0.244	416 (84.5)	17 (94.4)	0.250
	Yes	72 (15.7)	5 (9.6)		76 (15.5)	1 (5.6)	
**Resection category**	R0	341 (74.5)	41 (78.8)	0.489	368 (74.8)	14 (77.8)	0.774
	R1	117 (25.5)	11 (21.2)		124 (25.2)	4 (22.2)	
**Tumour regression grade** ^§^	TRG2	126 (49.6)	11 (52.4)	0.825	135 (50)	2 (40)	1.000
	TRG3	128 (50.4)	10 (47.6)		135 (50)	3 (60)	
	Total^§^	254 (100)	21 (100)		270 (100)	5 (100)	
**Neoadjuvant CTx**	No	204 (44.5)	31 (65.6)	0.039	222 (45.1)	13 (75.0)	0.023
	Yes	254 (55.5)	21 (34.4)		270 (54.9)	5 (25.0)	

* Only patients with EBV and MSI‐H‐negative tumours are included in the analysis.

^†^ Fisher's exact or chi‐squared test.

^‡^ TNM classification of malignant tumours according to the Seventh Edition of the UICC.

^**§**^ TRG corresponded only to patients with tumours treated with neoadjuvant CTx.

**Figure 3:**
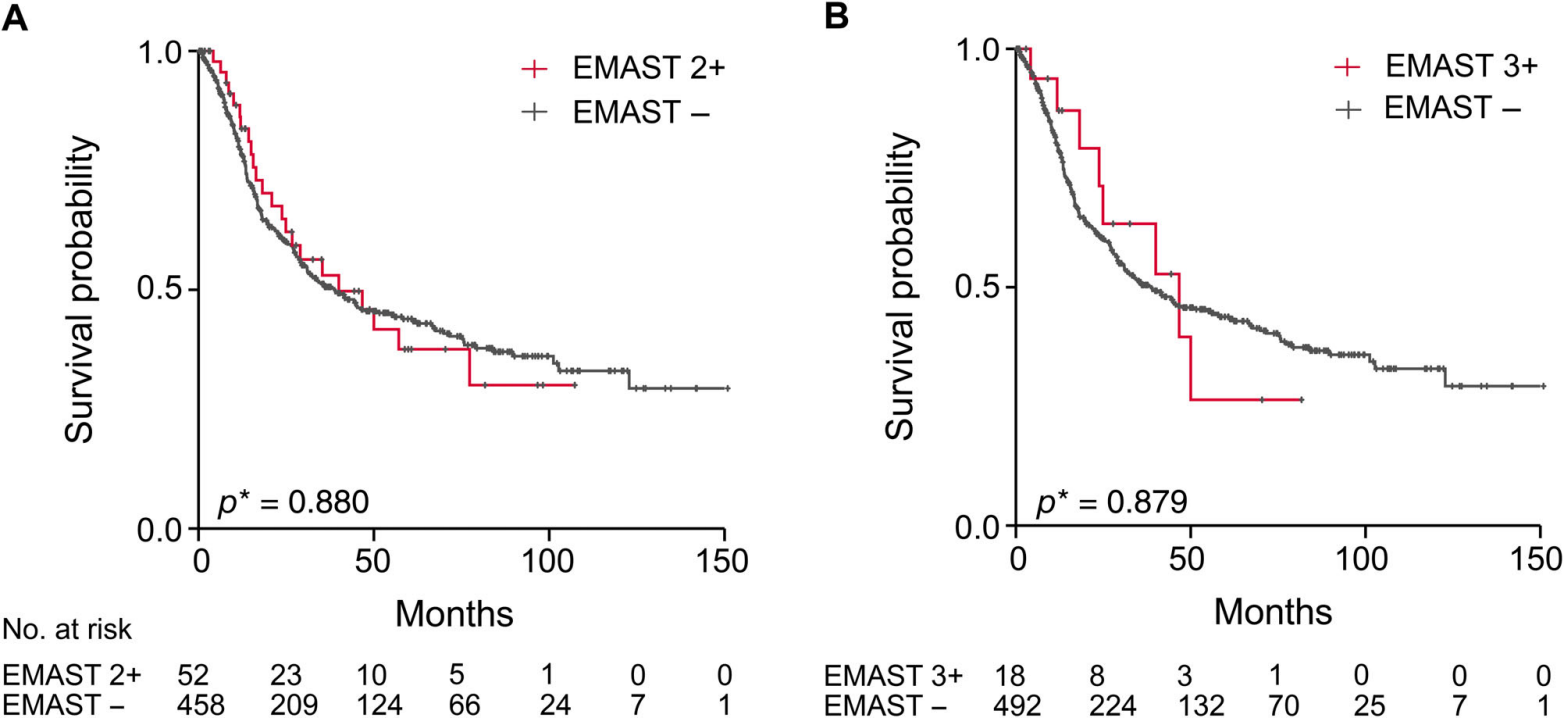
EMAST as molecular subtype and association with patient survival. Kaplan–Meier curves are shown for patients with EMAST‐negative (−) and EMAST‐positive (+) tumours according to (A) EMAST definition 2+ and (B) 3+. *Log‐rank test.

Regarding OS, no significant differences between EMAST (2+) or EMAST (3+) and the respective EMAST‐negative group were found. The median OS of EMAST (2+) patients was 40.0 months (95% confidence interval [CI] 16.4–63.6) compared to 38.7 months (95% CI 26.3–51.1) in the EMAST‐negative group (*p* = 0.880). In the EMAST (3+) category, the median OS was 46.7 months (95% CI 18.2–75.2) in the positive and 38.7 months (95% CI 26.2–51.2) in the negative group (*p* = 0.879). The Kaplan–Meier curves are shown in Figure 3.

Subgroup analysis of the patients stratified according to positive or negative lymph node status revealed, for patients with positive lymph nodes, a worse median OS for EMAST (2+) positive patients compared to the EMAST‐negative group (16.4 months, 95% CI 12.09–20.71 versus 25.5 months, 95% CI 18.74–32.26; *p* = 0.059). In the non‐CTx group, subgroup analysis of the patients stratified according to CTx yes/no showed a worse median OS for EMAST (2+) compared to EMAST‐negative patients (46.7 months, 95% CI 27.82–65.58 versus 70.0 months, 95% CI 33.34–106.67) and also for the EMAST (3+) compared to the respective EMAST‐negative group (46.7 months, 95% CI 15.98–77.42 versus 70 months, 95% CI 28.89–111.11). However, these differences were not statistically significant (*p* = 0.455 and *p* = 0.454, respectively).

### EMAST in pretherapeutic biopsies before neoadjuvant treatment and response to CTx

**Figure 4:**
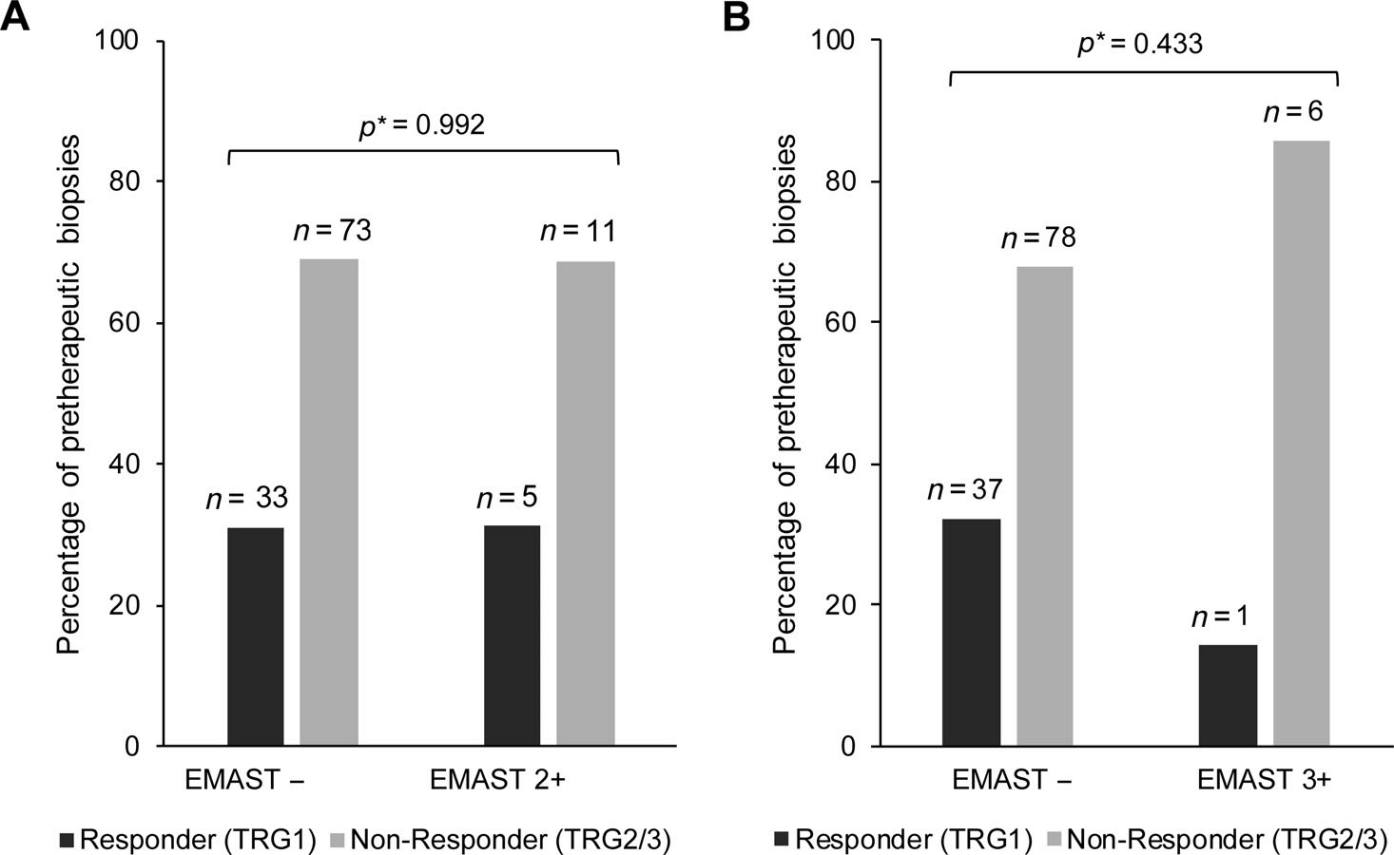
EMAST as molecular subtype and association with patient response to therapy. EMAST status of pretherapeutic biopsies and response to neoadjuvant CTx according to (A) EMAST definition 2+ and (B) 3+. MSI‐H and EBV (+) tumours are excluded from the analysis. *Fisher's exact or chi‐squared test.

Among the 142 analysed tumour biopsies before CTx, 31 (21.8%) showed EMAST (2+) and 22 (15.5%) showed EMAST (3+). Again, we excluded the MSI‐H (*n* = 15) and EBV+ (*n* = 5) tumours in this study cohort and compared EMAST positivity with response to CTx in overall 122 MSI‐H and EBV‐negative tumours (Figure 1). No association of EMAST (2+) with response was found, as 5 of 16 (31%) EMAST (2+) tumours were from responding patients compared to 33 of 106 (31%) in the EMAST‐negative group (*p* = 0.992) (Figure 4A). There were numerical, but no statistically significant, differences with respect to EMAST (3+) and response, as 1 of 7 EMAST 3+ (14%) tumours were from responding patients corresponding to 37 of 115 (32%) responders in the EMAST‐negative group (*p* = 0.433) (Figure 4B). In addition, only minor differences of EMAST‐positive and ‐negative tumours with respect to survival were found (EMAST 2+ and 3+: log‐rank *p* = 0.893 and *p* = 0.422, respectively).

## Discussion

In this study, we performed a comprehensive analysis of EMAST in a large GC cohort to clarify if EMAST alone or in the context of classical MSI represents a particular type of instability, which differentiates MSI‐H into specific subgroups and/or represents a unique type of instability associated with specific clinico‐pathological characteristics and patient survival or response to neoadjuvant CTx. Our results show nearly complete overlap between MSI‐H and EMAST positivity and indicate that EMAST alone is not a distinct instability type with distinctive clinico‐pathological characteristics.

The overall frequency of EMAST (2+) was, with 17.8% of the resected tumours showing these alterations, in a similar range to that reported in other studies of GC and also of colorectal carcinomas, which used a comparable definition for EMAST and microsatellite markers for EMAST analysis as in our study [18, 26].

Consideration of EMAST in the context of MSI showed that a very high percentage of MSI‐H tumours were also EMAST positive (96% for EMAST 2+ and 92.5% for EMAST 3+). Similar results were seen considering EMAST positivity in the context of MMR deficiency (MMRD) based on the expression analysis of the MMR proteins MLH1, MSH2, PMS2, and MSH6. For one of the two MSI‐H/EMAST‐negative tumours, immunohistochemical data were available and an isolated loss of MSH6 expression was found, which may be related to the negative instability pattern specifically observed at tetranucleotides in this tumour.

Thus, according to the nearly complete overlap of MSI‐H, determined by PCR‐based fragment analysis, and of MMRD, determined by immunohistochemistry, with EMAST‐positive tumours, our results indicate that differentiation into two distinct MSI‐H subtypes by EMAST seems to be very rare. The findings rather suggest that MMRD in the vast majority of these tumours is not restricted to instabilities at only mono or dinucleotide microsatellite repeats, but also affects tetranucleotide repeats. This is in line with several studies reporting a strong overlap between EMAST and MSI‐H [26, 27]. Furthermore, in an next generation sequencing (NGS)‐based analysis of EMAST in 248 colorectal tumours, all MSI tumours showed EMAST and, among the MSS tumours, some also demonstrated microsatellite mutations at tetranucleotide repeats, which however was not restricted to a specific subset of tumours, but was considered to represent stochastic events [28].

However, our results are in contrast to a study on GC by Fang *et al* [18], who showed EMAST positivity in only 59% of MSI‐H tumours and demonstrated genetic differences, such as a higher prevalence of mutations in DNA repair genes and of some clinico‐pathological features between EMAST+/MSI‐H and EMAST−/MSI‐H tumours, and a higher frequency of advanced tumour stages and worse survival in the EMAST−/MSI‐H group. Differences in the mutation pattern of several DNA repair genes, specific oncogenes, and tumour suppressor genes, and differences related to age and prognosis between the MSI‐H tumours with and without EMAST have also been described for patients with colorectal carcinoma [19, 20]. In addition, an overlap between EMAST and the MSI‐L phenotype has been demonstrated in colorectal cancer; however, this was not confirmed by our results [2, 16]. The reasons for these discrepancies may be manifold and may be related to the lack of a standardised determination and classification of EMAST and also related to the different methods used for the determination of conventional MSI. Furthermore, differences in the study populations regarding tumour stage or racial/ethnic disparities may play a role and EMAST has been reported to be twice as prevalent in African/Americans than in Caucasians [14].

Significantly lower nuclear MSH3 expression was observed in association with EMAST in our study. This is essentially in line with reports of reduced nuclear MSH3 expression in EMAST‐positive colorectal cancer [1, 29]. In contrast, no association of EMAST with MSH3 expression based on the determination of MSH3 by digital immunohistochemical analysis or by more conventional evaluation of MSH3 expression in colorectal or pancreatic carcinomas was described by others [13, 17, 30]. Thus, the role of MSH3 in EMAST is still a matter of debate.

With respect to the expression of the MMR proteins, MLH1, MSH2, MSH6, and PMS2, loss of one or two of these proteins was found in all MSI‐H/EMAST‐positive tumours with the majority of them demonstrating loss of the MLH1/PMS2 complex, which is in good accordance with numerous studies comparing MSI‐H determined by PCR and MMRD using immunohistochemistry [31, 32, 33]. Only a single EMAST‐negative and MSS tumour showed isolated loss of MSH6. Reduced or no MSI together with isolated MSH6 loss has been rarely observed in colorectal and endometrial carcinomas and has been attributed to partial impairment of MMR function [34, 35].

Considering EMAST alone as a distinct instability type or molecular subclass in the group of patients without MSI‐H and EBV+ tumours, we did not find clear associations of EMAST (2+) and (3+) positivity with specific clinico‐pathological characteristics, OS, or response of the patients to neoadjuvant CTx. A somewhat higher frequency of EMAST was found in patients not treated with CTx. One could speculate that this may reflect some difference in the sensitivity of EMAST‐positive tumours towards the applied chemotherapeutic agents. However, our results of the analysis of pretherapeutic biopsies before CTx, including patients with complete or nearly complete tumour regression in the resected specimen, do not support this view. Neither EMAST (2+) nor (3+) positivity demonstrated a significant association with response to neoadjuvant CTx. Several studies reported a specific link between EMAST and particular patient features. In colorectal cancer patients, EMAST was a negative prognostic factor for patients treated with 5‐FU‐based CTx, and a link between EMAST and an older age, a frailer phenotype, and worse prognosis was reported for colorectal cancer patients [29, 36]. In contrast, no prognostic difference between patients with EMAST‐positive and ‐negative tumours was found for pancreatic carcinomas or for colorectal cancer patients irrespective of treatment with adjuvant 5‐FU therapy [13, 37]. Thus, the results of these latter studies are essentially in line with our results. Again, the reasons for all these partially conflicting results may be related to the non‐standardised definition and determination of EMAST and differences in the study populations. A main reason, however, may be due to the fact that in some studies there is no concomitant analysis of MSI [29] or no clear separation of MSI‐H and EMAST positivity and hence no comparison of EMAST‐positive and ‐negative tumours in an MSS background [26, 27, 29, 38]. Thus, certain patient characteristics that are known for MSI‐H are attributed to EMAST, which reflects essentially the same deficiency of the MMR machinery encompassing MLH1, MSH2, MSH6, and PMS2.

A strength of our study is the large patient population, which enabled an analysis and a clear separation of EMAST positivity from classical MSI‐H, which in our opinion is a prerequisite for the investigation of whether EMAST represents a distinct instability type. We are aware, however, that this leads to small numbers in some subgroups and the results must be considered with care, as it cannot be excluded that EMAST positivity will demonstrate some effects in specific subgroups when analysing a higher number of patients. In particular, the fact that MSI‐H‐negative and EBV‐negative tumours are not a homogenous tumour group, but comprise different molecular subgroups, should be taken into account. A further limitation of our study is its retrospective nature and it has to be considered as an explorative analysis.

In conclusion, our results indicate that a considerable number of gastric cancer patients demonstrate EMAST in their tumours. There is nearly complete overlap of MSI‐H or aberrant expression of MLH1, MSH2, MSH6, or PMS2 with EMAST positivity and our findings do no indicate that EMAST differentiates MSI‐H tumours into two subgroups. Furthermore, our results suggest that EMAST alone is not a distinct instability type associated with prognosis or other relevant characteristics of GC patients.

## Author contributions statement

A‐LH, SW, MK and GK were involved in study design, data analysis, or interpretation. A‐LH and SW collected molecular data. MJ, JS‐H, AN, MMG, TS and WW contributed either pathological or clinical data. AH helped with statistical analysis. JS‐H, MJ, KS and BG were involved in immunohistochemical analysis. A‐LH and GK drafted the manuscript. All authors reviewed and approved the final submitted version.

## Supporting information


Supplementary materials and methods
**Table S1.** Chemotherapy regimens of the preoperatively treated patients included in the EMAST analysis
**Table S2.** Tetranucleotide microsatellite primer sequences
**Table S3.** Antibodies and their dilutions and manufacturers
**Table S4.** Association of EMAST/MSI status and expression of the MMR proteins (MLH1, PMS2, MSH2, MSH6)

## Data Availability

The data presented in this study are available in this article or supplementary material.
