# Supplementary material for: Elevated microsatellite instability at selected tetranucleotide (EMAST) repeats in gastric cancer: a distinct microsatellite instability type with potential clinical impact?
Source: J Pathol Clin Res. 2022 Jan 31;8(3):233–44. doi: 10.1002/cjp2.257 (PMC8977279; doi:10.1002/cjp2.257)
Supplement: Supplementary file 1 — Supplementary materials and methods Table S1. Chemotherapy regimens of the preoperatively treated patients included in the EMAST analysis Table S2. Tetranucleotide microsatellite primer sequences Table S3. Antibodies and their dilutions and manufacturers Table S4. Association of EMAST/MSI status and expression of the MMR proteins (MLH1, PMS2, MSH2, MSH6) [file CJP2-8-233-s001.docx]

**Elevated microsatellite instability at selected tetranucleotide repeats (EMAST) in gastric cancer: a distinct microsatellite instability type with potential clinical impact?**

A-L Herz *et al*. *J Pathol Clin Res* DOI: 10.1002/cjp2.257

**Supplementary material**

**Supplementary materials and methods**

**Table S1.** Chemotherapy regimens of the preoperatively treated patients included for the EMAST analysis

**Table S2.** Tetranucleotide microsatellite primer sequences

**Table S3.** Antibodies and their dilutions and manufacturer

**Table S4.** Association of EMAST/ MSI status and expression of the MMR proteins (MLH1, PMS2, MSH2, MSH6)

**Supplementary materials and methods**

**PCR conditions and fragment analysis for EMAST determination**

A multiplex PCR with fluorescence-tagged primers was performed using the Type-it Microsatellite PCR kit (Qiagen, Hilden, Germany). Non-tumour or tumour DNA was added to each PCR reaction in a final volume of 12.5µl. Cycle conditions were as follows: after an initial step of 95°C for 5 min, 32 cycles were performed consisting of denaturation at 95°C for 30 sec, annealing at 58°C for 90 sec and extension at 72°C for 30 sec and final extension at 60°C for 30 min. Separation and detection of the PCR products were performed using a 3130 Genetic Analyzer (Applied Biosystems, Foster City, CA) and the GeneMapper Software 5 (Applied Biosystem).

**Table S1.** Chemotherapy regimens of the preoperatively treated patients included for the EMAST analysis

| **Neoadjuvant chemotherapy** | **Resected tumours after CTx** | | **Tumour biopsies before CTx** | |
| --- | --- | --- | --- | --- |
|  | n | % | n | % |
| Total | 311 | 100 | 142 | 100 |
| Cis + 5-FU or Cap | 118 | 37,9 | 116 | 81,7 |
| Ox + 5-FU or Cap | 46 | 14,8 | 18 | 12,7 |
| Cis + 5-FU + Doc or Pac | 26 | 8,4 | 2 | 1,4 |
| Ox + 5-FU + Doc or Pac | 18 | 5,8 | 0 | 0 |
| Cis or Ox + 5-FU or Cap + Epi | 80 | 25,7 | 5 | 3,5 |
| Others | 23 | 7,4 | 1 | <1 |

Cis, cisplatin; Ox, oxaliplatin; 5-FU, 5-fluorouracil; Cap, capecitabine; Doc, docetaxel; Pac, paclitaxel; Epi, epirubicin; Others, combination of Cis/Ox with other agents as small molecule inhibitors or monoclonal antibodies (Imatinib, Panitumumab, Cetuximab) or cross over between different treatment regimens; No patient received preoperative radiotherapy; n/a, no data available.

**Table S2.** Tetranucleotide microsatellite primer sequences according to Devaraj et al. [14]

| EMAST Marker | Amplicon size [bp] | Range  [bp] | Forward primer | Reverse primer |
| --- | --- | --- | --- | --- |
| D20S85 | 156 | 138-154 | [FAM]GAGTATCCAGAGAGCTATTA | ATTACATGTTGAGACCCTG |
| MYCL1 | 181 | 140-209 | [ATO550]TGGCGAGACTCCATCAAAG | CTTTTTAAGCTGCAACAATTTC |
| D9S242 | 178 | 170-186 | [HEX]GTGAGAGTTCCTTCTGGC | ACTCCAGTACAAGACTCTG |
| D20S82 | 249 | 218-257 | [FAM]GCCTTGATCACACCACTACA | GTGGTCACTAAAGTTTCTGCT |
| D8S321 | 237 | 229-245 | [HEX]GATGAAAGAATGATAGATTACAG | ATCTTCTCATGCCATATCTGC |

**Table S3.** Antibodies and their dilutions and manufacturer

| **Primary antibody** | **Dilution** | **Manufacturer** |
| --- | --- | --- |
| MLH1 (M1) Mouse | prediluted (*Ready-to-use*) | Roche (Mannheim, Deutschland) |
| PMS2 (EPR3947) Rabbit | prediluted (*Ready-to-use*) | Cell Marque Corporation (Sierra, USA) |
| MSH2 (G219-1129) Mouse | 1:100 | Cell Marque Corporation (Sierra, USA) |
| MSH6 (610918) Mouse | 1:400 | BD Biosciences (New Jersey, USA) |
| MSH3[EPR4334(2)] Rabbit | 1:1500 | Abcam (Cambridge, England) |

**Table S4.** Association of EMAST/ MSI status and expression of the MMR proteins (MLH1, PMS2, MSH2, MSH6)

|  |  | | Loss of expression | | | | |
| --- | --- | --- | --- | --- | --- | --- | --- |
| MSI-status | | EMAST-status | | MLH1/  PMS2  n=43 | MSH2/  MSH6  n=1 | MSH6 n=2 | PMS2 n=1 |
| MSS+MSI-L | | EMAST - | | - | - | 1 | - |
|  | | EMAST 3+ | | - | - | - | - |
| MSI-H | | EMAST - | | 1 | - | 1 | - |
|  | | EMAST 3+ | | 42 | 1 | - | 1 |
| MSS+MSI-L | | EMAST - | | - | - | 1 | - |
|  | | EMAST 2+ | | - | - | - | - |
| MSI-H | | EMAST - | | - | - | 1 | - |
|  | | EMAST 2+ | | 43 | 1 | - | 1 |
